# Simultaneous Heart and Kidney Transplantation Using Circulatory Death Donors: Are Kidney Graft Outcomes Comparable With Brain Death Donors?

**DOI:** 10.1097/TXD.0000000000001853

**Published:** 2025-09-18

**Authors:** Sooyun Caroline Tavolacci, Kenji Okumura, Ameesh Isath, Gabriel B. Rodriguez, Corazon De La Pena, Junichi Shimamura, Ryosuke Misawa, Steven L. Lansman, Suguru Ohira

**Affiliations:** 1 Division of Cardiothoracic Surgery, Department of Surgery, Westchester Medical Center, Valhalla, NY.; 2 Department of Surgery, Research Institute, Westchester Medical Center, Valhalla, NY.; 3 Icahn School of Medicine at Mount Sinai, New York, NY.; 4 Department of Surgery, New York Medical College, Valhalla, NY.; 5 Department of Cardiology, Advanced Heart Disease and Transplant Cardiology, Brigham and Women’s Hospital, Boston, MA.

## Abstract

**Background.:**

There is limited evidence on heart and kidney transplants using donation after circulatory death (DCD) donors, especially regarding graft outcomes. However, little is known about the use of DCD donors for simultaneous heart and kidney transplants (SHKTs) compared with SHKTs using donation after brain death (DBD) donors.

**Methods.:**

From May 22, 2020, to September 30, 2023, 1129 adult patients received SHKTs (DCD, N = 91 versus DBD, N = 1038), identified using the United Network for Organ Sharing database, excluding other multiorgan transplants and retransplants. A propensity score matching was performed using characteristics. Ninety-one DCD- and 273 DBD-matched cases were compared.

**Results.:**

In the unmatched cohort, DCD recipients were older (DCD: 60 versus DBD: 58 y, *P* = 0.03), had a lower rate of dialysis at transplant (27% versus 40%, *P* = 0.03), and were of status 1 to 2 (43% versus 72%, *P* < 0.001). In the matched cohort, kidney delayed graft function (27% versus 22%, *P* = 0.29) was comparable, as were recipient survival (*P* = 0.19), heart graft survival (*P* = 0.19), and kidney graft survival (*P* = 0.17). In multivariate Cox proportional hazards analysis, donor type (DCD) was not associated with an increased risk of mortality (hazard ratio, 1.69; 95% confidence interval, 0.90-3.16; *P* = 0.10). Subgroup analysis showed that survival and freedom from graft failures were comparable between different modes of DCD recovery.

**Conclusions.:**

SHKT using DCD donors yields comparable survival and graft outcomes to those using DBD donors. These findings may help donor selection in heart transplant candidates with kidney dysfunction.

## Graphical abstract

**Figure:**
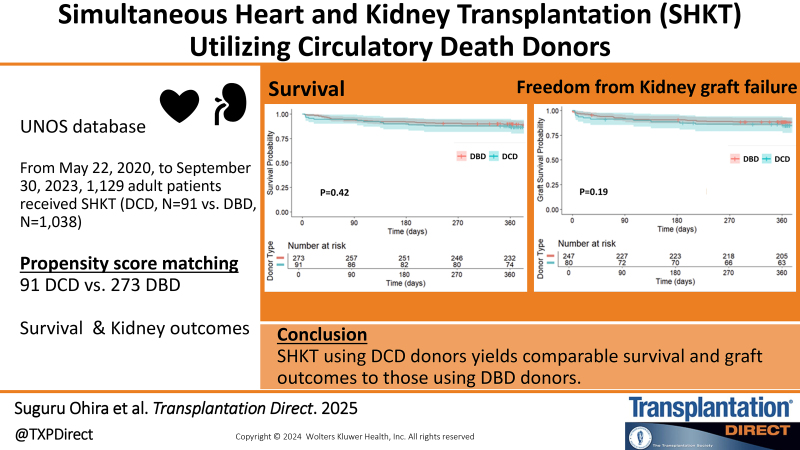

## INTRODUCTION

Pre– and post–heart transplant renal dysfunction is significantly associated with poor outcomes, especially in patients needing dialysis before orthotopic heart transplant (OHT).^1-3^ Simultaneous heart and kidney transplantation (SHKT) may improve survival and quality of life in this population by implanting 2 functional organs from a single donor, which also provides immunological advantages.^4,5^ The number of SHKT has continued to grow in the past 20 y due to a surge of end-stage heart failure patients with impaired renal function. Notably, this trend dramatically accelerated after the introduction of the new allocation policy on October 18, 2018, which prioritizes patients with acuity of illness.^4,6^ In this growing demand for SHKT, several important facts need to be taken into account despite its potential benefits. First, the utilization of 2 organs for 1 recipient may be controversial given the overall shortage of donors.^7^ Second, the complexity of 2 major surgeries and perioperative management may potentially increase the kidney graft failure rate and mortality compared with OHT alone.^8^ This can potentially compromise organ supply for isolated kidney transplant candidates for whom single-organ transplantation offers promising long-term survival and quality of life with a lower operative risk.^9^ Finally, a lower threshold of listing patients for dual-organ transplantation might be associated with an increase in “unnecessary” SHKT, where OHT alone could have led to the recovery of renal function through improvement of cardio-renal syndrome.^10^ One of the strategies to improve transplant outcomes and optimize organ utilization is the instrumentation of the new kidney safety net policy. This policy, launched in June 2023, allows OHT recipients who meet the eligibility criteria for a kidney transplant to receive additional priority in kidney matches within 60–365 d after OHT.^2,3,11^

Another approach to increase the opportunity of SHKT is by using donation after circulatory death (DCD) donors.^12,13^ The important difference between DBD and DCD procurement is the presence of a warm ischemic period before DCD procurement in comparison with donation after brain death (DBD) donors recovery, which can potentially impact organ function, especially in the setting of multiorgan transplant. Although the initial outcomes of multiorgan DCD transplant seem promising in unmatched comparisons, clinical evidence of SHKT using DCD donors remains limited, especially the detailed kidney graft outcomes. A better understanding of overall and kidney graft outcomes of DCD-SHKT may help elucidate its indication and barriers to the expanding donor pool. This study aims to analyze the outcomes of SKHT using DCD versus DBD recovery using propensity score (PS) matching. Our hypothesis was that DCD-SHKT provides comparable survival and graft outcomes.

## MATERIALS AND METHODS

### Study Population

The United Network for Organ Sharing (UNOS) database was queried from May 22, 2020, to September 30, 2023, as the first DCD-SHKT was performed on May 22, 2020. A total of 1129 SHKTs in adult patients, excluding other multiorgan transplants (eg, heart-liver-kidney, heart-lung-kidney transplants) and retransplants, were identified. Recipients younger than 18 y were also excluded. Of the 1129 patients identified, 1038 received DBD-SHKT, and 91 received DCD-SHKT. A 1:3 ratio PS matching was performed: DCD (N = 91) and DBD (N = 273). We merged the outcomes of heart and kidney recipients to analyze kidney outcomes, including kidney delayed graft function (KDGF) and kidney graft survival. The most recent follow-up date was on March 31, 2024. This study was approved by the New York Medical College Institutional Review Board (No. 14680).

### Endpoints and Definitions

All definitions of variables are described on the UNOS web site (https://unos.org/data/data-collection/). Ischemic time is defined as the duration from cross-clamping to reperfusion. The different recovery methods in DCD-SHKT: direct procurement and perfusion (DPP) versus normothermic regional perfusion (NRP) were defined as follows: the interval from brain death time to aortic clamp time was used to categorize the recovery method, similar to previous studies,^14-16^ which showed a bimodal distribution with the minor mode at 20 min (**Figure S1, SDC,** https://links.lww.com/TXD/A785). Based on this distribution, an interval of <20 min from brain death to aortic clamp time was classified as DPP, whereas an interval of ≥20 min was classified as NRP.

The primary outcomes were recipient and kidney graft survival, as well as KDGF, defined as the requirement for hemodialysis within the first week after kidney transplant. UNOS defines heart or kidney graft failure as 1 of the following: removal of the transplanted organ (eg, retransplant), recipient death, or placement of the recipient on a chronic allograft support system (eg, hemodialysis for kidney transplant). Secondary outcomes included rates of stroke, acute rejection, and dialysis. The estimated posttransplant survival (EPTS) score of kidney recipients was calculated using 4 factors: age, current diagnosis of diabetes, prior solid organ transplants, and time on dialysis.^17^ The kidney donor profile index (KDPI) of donors was calculated using 10 variables that influence donor organ quality: age, height, weight, cause of death, last serum creatinine, history of diabetes, hypertension, hepatitis C serostatus, ethnicity, and whether the donation was after DCD versus DBD.^18^ For both EPTS and KDPI, lower values predict a higher chance of kidney graft survival.

### Statistical Analysis

Baseline characteristics and outcomes were reported as median with interquartile range for continuous variables or number and percentage for categorical variables. The Wilcoxon rank-sum test, Pearson’s chi-square test, and Fisher exact test were used for group comparison depending on variable types and distributions. Considering the significantly small number of DCD cases, we used a 1:3 PS matching with the nearest neighbor using 17 recipient characteristics (age, gender, ethnicity/race, diabetes, body mass index [BMI], smoking, creatinine, antibiotics use within 2 wk prior to transplant, ischemic cardiomyopathy, use of Impella, durable left ventricular assist device [LVAD] and intra-aortic balloon pump, ventilator support, extracorporeal membrane oxygenation [ECMO], prior cardiac surgery, end status, and blood type) and 7 donor characteristics (age, gender, ethnicity/race, BMI, creatinine, cause of death, and left ventricular ejection fraction). Covariates were selected on the basis of clinical relevance and previous literature as described earlier. Patients with missing data were excluded from PS matching. After matching, all covariates had a standardized difference of ≤10%, which was considered a threshold for ideal balance between the 2 cohorts (**Figure S2, SDC,** https://links.lww.com/TXD/A785).

Overall survival curves were derived using the Kaplan-Meier method; differences between cohorts were assessed using the log-rank test. Adjusted posttransplant survival was modeled using multivariate Cox proportional hazards regression. Factors identified as clinically relevant based on previous literature or clinical knowledge were included in the multivariable regression analysis. The results were expressed as hazard ratios (HRs) with 95% confidence intervals (CIs). All tests were 2-tailed, and a *P* value of <0.05 was considered significant. All statistical analyses were performed using RStudio version 4.3.2.

## RESULTS

### Baseline Characteristics

**FIGURE 1.**
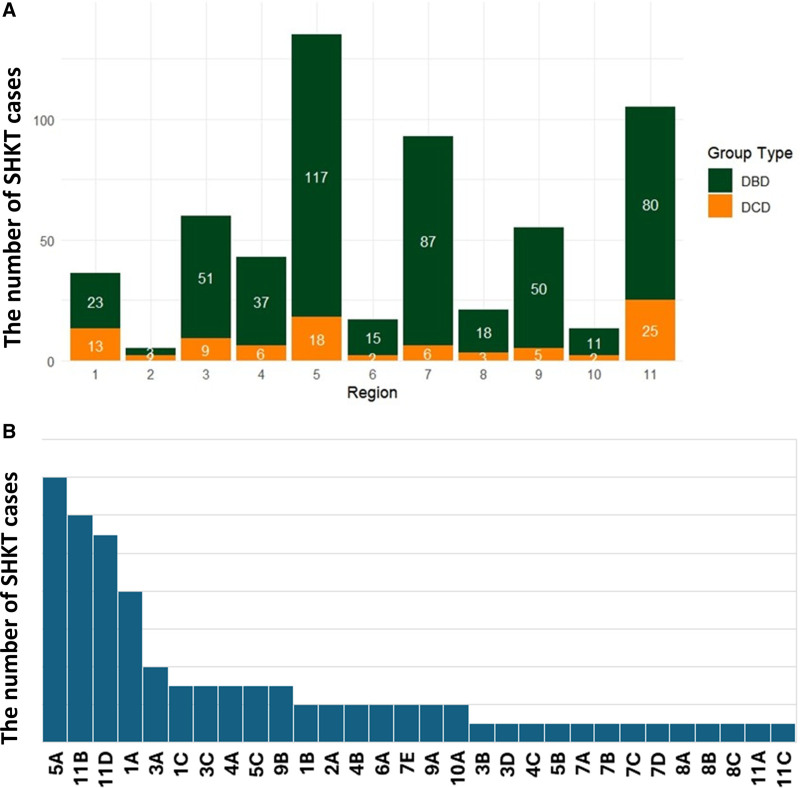
A, Regional DBD and DCD cases at centers performing both DCD- and DBD-SKHT. UNOS regions; Connecticut, Eastern Vermont, Maine, Massachusetts, New Hampshire, and Rhode Island, 2; Delaware, District of Columbia, Maryland, New Jersey, Pennsylvania, and West Virginia, 3; Alabama, Arkansas, Florida, Georgia, Louisiana, Mississippi, and Puerto Rico, 4; Oklahoma and Texas, 5; Arizona, California, Nevada, New Mexico, and Utah, 6; Alaska, Hawaii, Idaho, Montana, Oregon, and Washington, 7; Illinois, Minnesota, North Dakota, South Dakota, and Wisconsin, 8; Colorado, Iowa, Kansas, Missouri, Nebraska, and Wyoming, 9; New York and Western Vermont, 10; Indiana, Michigan, and Ohio, 11; Kentucky, North Carolina, South Carolina, Tennessee, and Virginia (https://unos.org/community/regions/). B, DCD_SHKT Cases by Centers from May 22, 2020, to September 30, 2023. Encrypted Center Code was replaced by region and alphabet (eg, region 1 center A—1A, region 3 center C—3C), also shown in **Table S2** (**SDC,** https://links.lww.com/TXD/A785). DBD, donation after brain death; DCD, donation after circulatory death; SHKT, simultaneous heart and kidney transplant.

During the study period, 51.6% of all SHKTs (N = 583/1129) were performed at 30 centers performing both DCD- and DBD-SKHT where DCD-SHKT accounted for 15.6% (N = 91/583) of their SHKT cases (Figure 1A). Most centers only performed 1 case of DCD-SHKT, whereas some centers that performed ≥4 cases showed a high proportion DCD-SHKT per all SHKTs performed at the center (Figure 1B; **Table S1, SDC,** https://links.lww.com/TXD/A785). Each UNOS region has at least 1 center that has performed both DCD- and DBD-SHKT. Regions 5 and 11 performed 24% and 16% of total national DBD-SHKTs and 20% and 27% of national DCD-SHKTs, respectively.

In the unmatched cohort, the DCD group had older recipients (DCD, 60 [53–65.5] versus DBD, 58 [50.3–64] y, *P* = 0.03), lower prevalence of dialysis at the time of SHKT (27% versus 40%, *P* = 0.03), and a lower rate of status 1 to 2 patients (43% versus 72%, *P *< 0.001; Table 1). Recipients’ EPTS score (DCD, 41% [28–51] versus DBD, 36% [22–50], *P* = 0.04) was higher in the DCD group (**Figure S3, SDC,** https://links.lww.com/TXD/A785). Donors were younger (30 [24–35] versus 32 [25–39] y, *P* = 0.02) and predominantly of White race (74% versus 59%, *P* = 0.01) in the DCD group. The KDPI score was higher in the DCD group (22% [13–32] versus 18% [7–32], *P* = 0.04; **Figure S3, SDC,** https://links.lww.com/TXD/A785). The distance from donor to recipient hospital was longer in the DCD group (344 [153.5–581.5] versus 231.5 [92–390.8] miles, *P* < 0.001), as was heart ischemic time (4.9 [3.4–6.4] versus 3.5 [2.9–4] h, *P* < 0.001), and kidney ischemic time (19.2 [13.7–24.8] versus 16.3 [9.3–22] h, *P* = 0.002). Days on the waiting list were comparable for both heart (42 [9.5–160.5] versus 33 [11–107.8] d, *P* = 0.42) and kidney (37 [10–164] versus 29 [10–91] d, *P* = 0.15) transplants.

**TABLE 1.** Baseline recipient, donor, and organ procurement characteristics of unmatched and matched cohorts

Variable	DCD-SHKT (N = 91)	DBD-SHKT (N = 1038)	*P*	Matched DBD-SHKT (N = 273)	*P*
Recipient					
Age, y	60.0 (53.0–65.5)	58.0 (50.3–64.0)	0.032	59.0 (52.0–64.0)	0.36
Sex					
Male	74 (81%)	832 (80%)	0.90	214 (78%)	0.55
Race			0.56		0.81
Non-White	55 (60%)	588 (57%)		161 (59%)	
White	36 (40%)	450 (43%)		112 (41%)	
Diabetes	50 (55%)	485 (47%)	0.16	144 (53%)	0.72
BMI, kg/m^2^	27.0 (24.0–31.6)	27.2 (23.9–31.0)	0.96	27.0 (23.8–30.4)	0.60
Dialysis at transplant	25 (27%)	415 (40%)	0.025	98 (36%)	0.14
History of smoking	36 (40%)	413 (40%)	>0.99	107 (39%)	0.95
Cerebrovascular disease	8 (8.8%)	96 (9.2%)	>0.99	26 (9.5%)	0.84
IV antibiotics therapy	6 (6.6%)	159 (15%)	0.035	19 (7.0%)	0.90
Status			<0.001		0.47
Status 1–2	39 (43%)	751 (72%)		129 (47%)	
Status 3–6	52 (57%)	287 (28%)		144 (53%)	
Primary diagnosis			0.54		0.50
Ischemic CM	28 (31%)	359 (35%)		74 (27%)	
Nonischemic CM	63 (69%)	679 (65%)		199 (73%)	
Durable LVAD	17 (19%)	138 (13%)	0.20	58 (21%)	0.60
IABP	8 (8.8%)	285 (27%)	<0.001	27 (9.9%)	0.76
Impella	1 (1.1%)	70 (6.7%)	0.057	3 (1.1%)	>0.99
Ventilator	2 (2.2%)	21 (2.0%)	>0.99	6 (2.2%)	>0.99
ECMO	5 (5.5%)	58 (5.6%)	>0.99	16 (5.9%)	0.90
History of cardiac surgery	31 (34%)	346 (33%)	0.98	92 (34%)	0.95
Initial EPTS score	41 (28%–51%)	36 (22%–50%)	0.042	39 (26%–54%)	0.62
Recipient ABO			0.35		0.99
A	30 (33%)	395 (38%)		93 (34%)	
AB	4 (4.4%)	56 (5.4%)		12 (4.4%)	
B	12 (13%)	174 (17%)		33 (12%)	
O	45 (49%)	413 (40%)		135 (49%)	
Donor					
Age	30.0 (24.0–35.0)	32.0 (25.0–39.0)	0.015	28.0 (21.0–37.0)	0.64
Sex					
Male	76 (84%)	801 (77%)	0.21	220 (81%)	0.53
Race			0.010		0.95
Non-White	24 (26%)	423 (41%)		73 (27%)	
White	67 (74%)	615 (59%)		200 (73%)	
BMI, kg/m^2^	27.1 (24.3–31.6)	27.1 (23.6–31.1)	0.52	26.2 (23.0–31.2)	0.25
Creatinine, mg/dL	0.7 (0.6-1.0)	0.9 (0.7–1.2)	<0.001	0.8 (0.6–1.0)	0.24
Cause of death			0.17		0.72
Head trauma	46 (51%)	441 (42%)		132 (48%)	
Other	45 (49%)	597 (58%)		141 (52%)	
LVEF, %	64.0 (60.0–67.0)	60.0 (57.0–65.0)	0.13	63.0 (60.0–66.0)	0.85
KDPI score, %	22 (13%–32%)	18 (7%–32%)	0.04	11 (4%–24%)	<0.001
ABO match			0.82		0.11
Identical	79 (87%)	886 (85%)		252 (92%)	
Compatible	12 (13%)	152 (15%)		21 (7.7%)	
Organ					
Heart procurement distance, miles	344.0 (153.5–581.5)	231.5 (92.0–390.8)	<0.001	212.0 (92.0–366.0)	<0.001
Heart waiting list time, d	42.0 (9.5–160.5)	33.0 (11.0–107.8)	0.42	52.0 (14.0–188.0)	0.26
Heart ischemic time, h	4.9 (3.4–6.4)	3.5 (2.9–4.0)	<0.001	3.5 (2.9–4.0)	<0.001
Heart machine perfusion	53/91 (58%)	44/1038 (4.2%)	<0.001	8/273 (2.9%)	<0.001
Kidney cold ischemic time, h	19.2 (13.7–24.8)	16.3 (9.3–22.0)	0.002	16.6 (9.9–21.5)	0.002
Kidney waiting list time, d	37.0 (10.0–164.0)	29.0 (10.0–91.0)	0.15	43.0 (13.0–131.3)	0.98

BMI, body mass index; CM, cardiomyopathy; DBD, donation after brain death; DCD, donation after circulatory death; ECMO, extracorporeal membrane oxygenation; EPTS, estimated posttransplant survival; IABP; intra-aortic balloon pump; IV, intravenous; KDPI, Kidney Donor Profile Index; LVAD, left ventricular assist device; LVEF, left ventricular ejection fraction; SHKT, simultaneous heart and kidney transplant.

Heart ischemic time is defined as the period from donor death to reperfusion in the recipient, including both true ischemia time and ex vivo perfusion or normothermic regional perfusion time.

After PS matching, there was no difference in recipient and donor characteristics between DCD versus DBD-SHKT, including dialysis and the listing status at the time of SHKT (Table 1), except for donor KDPI (DCD, 22% versus DBD, 11%, *P* < 0.001). Days on the waitlist were comparable between DCD (42.0 d) and DBD (52.0 d; *P* = 0.26). The distance of procurement (344 [153.5–581.5] versus 212 [92–366] miles, *P* < 0.001), heart ischemic time (4.9 [3.4–6.4] versus 3.5 [2.9–4] h, *P* < 0.001), and kidney cold ischemic time (19.2 [13.7–24.8] versus 16.6 [9.9–21.5] h, *P* = 0.002) were longer in the DCD group.

### Transplant Outcomes

**FIGURE 2.**
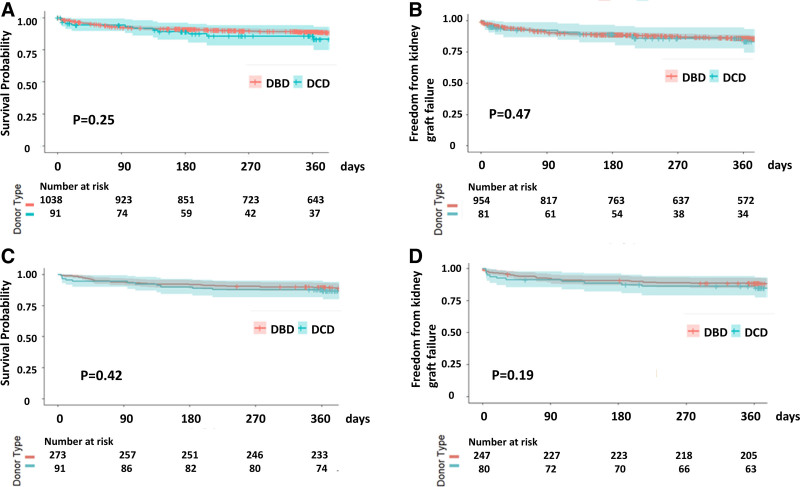
Recipient and graft survival outcome in unmatched and matched cohorts. Unmatched cohort (A and B) and matched cohort (C and D). A and C, Recipient survival. B and D, Kidney graft survival. DBD, donation after brain death; DCD, donation after circulatory death.

The incidence of KDGF was comparable in both nonmatched (DCD, 27% versus DBD, 26%, *P* = 0.68) and matched cohorts (DCD, 27% versus DBD, 22%, *P *= 0.29), whereas the creatinine level at discharge was higher in the DCD group (DCD, 1.6 [1.2–2.4] versus matched DBD, 1.2 [0.9–1.7] mg/dL, *P* < 0.001; Table 2). There was no significant difference in the incidence of acute rejection, dialysis, stroke, pacemaker placement, and length of stay. Recipient survival (median follow-up: 366.5 [182.75–730] d, *P *= 0.19), freedom from heart graft failure (*P* = 0.19), and kidney graft failure (*P* = 0.17) were similar in both nonmatched and matched cohorts (Figure 2). In the matched group, 1-y survival was 87% in the DCD-SHKT group and 90.8% in the DBD-SHKT group (*P* = 0.37). Multivariate Cox proportional hazards analysis showed that donor type (DCD) was not associated with an increased risk of mortality (HR, 1.69; 95% CI, 0.90-3.16; *P *= 0.10; **Table S2, SDC,** https://links.lww.com/TXD/A785). Recipient age, female recipient, and dialysis at the time of SHKT were identified as predictors of mortality.

**TABLE 2.** Heart and kidney transplant outcome of unmatched and matched cohorts

Variable	DCD-SHKT (N = 91)	DBD-SHKT (N = 1038)	*P*	Matched DBD-SHKT (N = 273)	*P*
Acute rejection, heart	6 (6.6%)	89 (8.6%)	0.65	14 (5.1%)	0.79
Dialysis	34 (37%)	365 (35%)	0.77	85 (31%)	0.33
Stroke	2 (2.2%)	44 (4.3%)	0.50	7 (2.6%)	>0.99
Pacemaker	2 (2.2%)	14 (1.4%)	0.85	3 (1.1%)	0.79
Kidney delayed graft function	25 (27%)	274 (26%)	0.68	61 (22%)	0.29
Length of stay, d	20.0 (15.0–34.0)	22.0 (15.0–33.0)	0.73	21.0 (15.0–33.0)	0.97
Patient mortality at 30 d	5 (5.5%)	35 (3.4%)	0.45	6 (2.2%)	0.22
Patient mortality at 6 mo	9 (9.9%)	91 (8.8%)	0.87	19 (7.0%)	0.50
Patient mortality at 1 y	12 (13%)	108 (10%)	0.52	25 (9.2%)	0.37
Heart graft failure at 6 mo	9 (9.9%)	92 (8.9%)	0.89	19 (7.0%)	0.50
Heart graft failure at 1 y	12 (13%)	109 (11%)	0.54	25 (9.2%)	0.37
Kidney graft failure at 6 mo	8 (9.9%)	103 (11%)	0.94	19 (7.8%)	0.71
Kidney graft failure at 1 y	11 (14%)	123 (13%)	>0.99	24 (9.8%)	0.46
Kidney acute rejection before discharge	1 (1.2%)	10 (1.1%)	>0.99	4 (1.7%)	>0.99
Unknown	10 (11%)	84 (8.1%)		28 (10%)	
Resume dialysis maintenance after kidney transplant	1 (1.1%)	33 (3.2%)	0.43	7 (2.6%)	0.68
Recipient creatinine at discharge, mg/dL	1.6 (1.2–2.4)	1.3 (0.9–1.8)	<0.001	1.2 (0.9–1.7)	<0.001

DBD, donation after brain death; DCD, donation after circulatory death; SHKT, simultaneous heart and kidney transplant.

The impact of KDGF on transplant outcomes was analyzed within the DCD group. Initial EPTS and KDPI scores, as well as heart ischemic time and kidney cold ischemic time, were not significantly different. Recipients who experienced KDGF had worse kidney graft survival (KDGF group: 72% versus no KDGF group: 92.9%, *P* = 0.029) and worse overall survival (72% versus 94.6%, *P* = 0.013) at 1 y (Table 3).

**TABLE 3.** Characteristics and transplant outcomes of the DCD group by kidney delayed graft function

Variable	KDGF (N = 25)	No KDGF (N = 56)	*P*
Initial EPTS score	0.5 (0.3–0.5)	0.4 (0.3–0.5)	0.11
KDPI score	0.3 (0.1–0.4)	0.2 (0.1–0.3)	0.60
Days in waiting list, d	43.0 (13.0–83.0)	40.0 (6.8–227.0)	>0.99
Distance, miles	408.0 (269.0–631.0)	265.0 (126.8–528.3)	0.078
Ischemic time, h	5.5 (3.9–6.7)	4.7 (3.3–6.1)	0.11
Kidney waiting list time, d	32.0 (12.0–57.0)	42.0 (9.5–261.5)	0.30
Kidney cold ischemic time, h	17.4 (13.0–22.4)	20.1 (15.3–25.2)	0.29
Machine perfusion	15/25 (60%)	32/56 (57%)	>0.99
Acute rejection	2/25 (8.0%)	3/56 (5.4%)	>0.99
Stroke	0/25 (0%)	1/56 (1.8%)	>0.99
Pacemaker	0/25 (0%)	2/56 (3.6%)	0.86
Length of stay, d	31.0 (19.5–47.0)	18.0 (13.0–26.3)	0.030
Patient mortality at 30 d	4/25 (16%)	1/56 (1.8%)	0.050
Patient mortality at 6 mo	5/25 (20%)	2/56 (3.6%)	0.045
Patient mortality at 1 y	7/25 (28%)	3/56 (5.4%)	0.013
Graft failure at 6 mo	5/25 (20%)	2/56 (3.6%)	0.045
Graft failure at 1 y	7/25 (28%)	3/56 (5.4%)	0.013
Kidney graft failure at 6 mo	5/25 (20%)	3/56 (5.4%)	0.10
Kidney graft failure at 1 y	7/25 (28%)	4/56 (7.1%)	0.029
Recipient creatinine at discharge (mg/dL)	1.8 (1.3–3.7)	1.6 (1.1–2.4)	0.22

DCD, donation after circulatory death; EPTS, estimated posttransplant survival; KDPI, Kidney Donor Profile Index.

### Different DCD Recovery Methods: DPP Versus NRP

**FIGURE 3.**
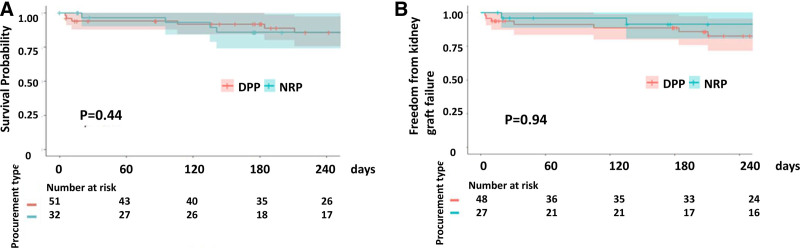
Recipient survival (A) and kidney graft survival (B) outcomes in subanalysis of the DCD cohort by a procurement method. DPP, direct procurement and perfusion; NRP, normothermic regional perfusion.

In the DCD group with available ischemic time criteria, 51 patients had DPP, and 32 patients received NRP. Donors in the NRP group were older (DPP, 28 [21–34] versus NRP, 33 [28.5–39] y, *P* = 0.002), and more likely men (76% versus 97%, *P* = 0.013; **Table S3, SDC,** https://links.lww.com/TXD/A785). The distance of procurement was longer in the DPP group (400 [188–619] versus 222.5 [59.3-392.3] miles, *P* = 0.021). Recipients’ EPTS and donor KDPI score were higher in the NRP group. Although serum creatinine level at the time of discharge was higher in the DPP group (DPP, 2.0 versus NRP, 1.3 mg/dL, *P *= 0.008), there was no difference in the rate of KDGF, requirement of dialysis (**Table S3, SDC,** https://links.lww.com/TXD/A785), kidney graft survival (*P* = 0.94), and overall recipient survival (*P* = 0.44; Figure 3).

### Transplant Centers Performing Both DCD- and DBD-SKHT

Among centers performing both DCD- and DBD-SHKT, the usage of durable LVAD was higher in the DCD group (DCD, 19% versus DBD, 9.4%, *P* = 0.014; **Table S4, SDC,** https://links.lww.com/TXD/A785). The rate of dialysis at transplant and UNOS status 1 or 2 at transplant were higher in DBD recipients. The incidence of KDGF was similar (27% versus 27%, *P *= 0.81); however, recipient serum creatinine at discharge was higher in the DCD group (1.6 [1.2–2.4] versus 1.3 [0.9–1.9] mg/dL, *P* < 0.001). Recipients’ survival (*P* = 0.069) and freedom from kidney graft failure (*P* = 0.41) were not different between DCD- and DBD-SHKT (**Figure S4, SDC,** https://links.lww.com/TXD/A785).

### Transplant Centers Performing Both DCD- and DBD-SKHT Versus Centers Performing only DBD-SKHT

When comparing the centers performing both DCD- and DBD-SHKT (DCD-SHKT centers) with the centers performing only DBD-SHKT (non-DCD-SHKT centers), there was no difference in baseline characteristics except for a higher percentage of status 3–6 (DCD-SHKT centers: 36% versus Non-DCD-SHKT centers: 23%, *P* < 0.001), lower usage of durable LVAD (11% versus 17%, *P* = 0.004) and Impella (4.5% versus 8.2%, *P* = 0.013), and a higher KDPI score (21% [9–35] versus 16% [7–28], *P* < 0.001) in centers A (**Table S5, SDC,** https://links.lww.com/TXD/A785). Days on the heart waiting list (29 [9–90.5] versus 39 [14–144.3] d, *P* < 0.001) and kidney waiting list (24 [8–87.3] versus 35 [12–111] d, *P* < 0.001) were significantly shorter in DCD-SHKT centers. The incidence of KDGF was comparable (DCD-SHKT centers, 27% versus non-DCD-SHKT centers, 26%, *P *= 0.54), as was freedom from kidney (89% versus 85%, *P* = 0.10) graft failure (**Figure S5, SDC,** https://links.lww.com/TXD/A785). Multivariable Cox proportional hazards analysis showed that non-DCD-SHKT centers are not associated with a higher risk of recipient mortality (HR, 1.26; 95% CI, 0.91-1.974; *P* = 0.172).

## DISCUSSION

### Survival and Graft Outcomes of DCD-SHKT

Given that the number of DBD transplants has plateaued recently,^19^ DCD donors could play a crucial role in not only shortening waitlist duration in patients with high acuity of illness but also increasing opportunities for candidates with expected prolonged waiting time, such as those with a durable LVAD, blood type O, or high BMI. We observed some differences in baseline characteristics between DCD-SHKT and DBD-SHKT in the nonmatched cohort. This aligns with prior reports of isolated DCD heart transplants, which showed that DCD groups had more patients with status 3–6, a numerically higher rate of durable LVAD use, and more blood type O recipients.^20,21^ The comparable transplant outcomes between DCD-SHKT and DBD-SHKT observed in this study are notably important given the increasing waitlist times, even for high-priority (status 2) patients, who constitute the majority under the current allocation policy.^22^ Overall, the primary analysis using PS matching supports the use of DCD donors to enhance waitlist outcomes without negatively impacting overall transplant outcomes.

In general, most centers prefer younger donors for SHKT, resulting in a lower KDPI (around 20%),^6^ which is significantly lower than that of isolated kidney transplant, where the KDPI is 43%.^9^ In our study, the KDPI was higher in the DCD group despite the younger donor age. This could be due to “DCD donor” being 1 of the variables used to calculate the KDPI, thereby increasing KDPI itself. Although the creatinine level at discharge was higher in DCD recipients, possibly reflecting the impact of warm ischemia on visceral organ function, the rates of KDGF, dialysis rate during hospitalization, and overall or graft survival were comparable.^12,13,15,20,21^ KDGF is a temporary event but is known as a significant prognostic marker after DBD-SHKT for not only renal outcomes but also for overall survival.^23^ DCD-SHKT recipients who experienced KDGF showed the worst outcome compared with to those who did not. Notably, we performed an additional logistic regression analysis, where DCD was not associated with KDGF (odds ratio, 1.07; 95% CI, 0.64-1.74; *P* = 0.80). The incidence of KDGF and its impact on long-term outcomes warrants further investigation in the context of DCD-SHKT as prior studies have shown that isolated kidney transplants using DCD donors had a higher KDGF rate compared with those using DBD donors.^24^

### Different Types of DCD Recovery Methods in SHKT

Recent studies on DCD heart transplants have indicated that NRP and DPP procurements are associated with similar 1-y post-OHT survival rates.^16,25,26^ A major concern with NRP is its ethical implications, and hence, it is not universally available or allowed.^16,25^ DPP, on the other hand, may raise concerns regarding the need for donor blood collection for ex vivo normothermic perfusion prior to aortic cross-clamping.^26^ This process can lead to prolonged warm ischemia of the abdominal organs and lungs, potentially resulting in worse transplant outcomes or a higher rate of organ discard of liver gaft.^16,26,27^ Although kidneys are more tolerant of ischemia compared with liver grafts,^26,28,29^ additional nephrectomy time can increase the risk of graft dysfunction in kidneys from DCD donors. In subgroup analysis, serum creatinine levels at discharge were higher in the DPP group than in the NRP group although the kidney graft outcomes were comparable. More evidence is warranted, as different DCD recovery methods represent an important area of interest that could potentially impact graft outcomes.^16,17^

### Transplant Centers Performing DCD-SHKT

Among these centers performing both DCD- and DBD-SHKT, a higher proportion of status 3–6 patients received DCD-SHKT, which correlated with a higher rate of durable LVAD use and a great number of blood type O recipients (**Table S3, SDC,** https://links.lww.com/TXD/A785). This may suggest that DCD donors are being effectively used to improve the gap between organ demand and donor supply in those centers. Although not statistically significant, survival in patients who received the DCD-SHKT at those DCD centers was numerically lower than in patients who received the DBD-SHKT at their centers (**Figure S4, SDC,** https://links.lww.com/TXD/A785). This finding warrants further investigation with greater statistical power. Finally, centers performing both DCD- and DBD-SHKT demonstrated significantly shorter waitlist times with similar outcomes compared with centers that performed only DBD-SHKT. Previous studies reported that patients who spend twice as long on the waitlist have a 10% higher chance of transplant failure.^30^ In this regard, the use of DCD donors for SHKT can potentially reduce waitlist times and improve waitlist outcomes, particularly for patients with limited treatment options in the era of organ shortages.^31^

### The New Kidney Safety Net Policy

The new kidney safety net policy was launched on June 29, 2023. We were unable to analyze its impact as our study period barely included recipients who were transplanted after the implementation. This strategy is an important option in patients with impaired renal function because a certain number of patients are not able to proceed to SHKT due to hemodynamic instability after heart transplantation, such as primary graft dysfunction requiring VA-ECMO, major bleeding, or requirement of high-dose vasopressor or inotropes. In this scenario, considerable risks of SHKT may outweigh its benefits, with a possibility of developing a nonfunctioning kidney graft. Therefore, not proceeding with simultaneous kidney transplantation not only benefits the SHKT candidate but also allows the reallocation of a kidney graft to other kidney graft recipients with a high success rate.^9^

### Limitations

Several limitations should be noted. First, this is a retrospective study that relied on national registry data, meaning that unmeasured variables in the database could influence the outcomes. The number of donor variables is limited in the UNOS database. Additionally, there were some missing data. The follow-up period for the DCD group is limited because of the relatively short history of DCD heart transplants. Outcomes may differ with a larger sample size and a longer follow-up period. It is also possible that more transplant centers are now performing both DCD- and DBD-SHKT. Also, the database lacks variables to determine the duration of pretransplant dialysis in heart and kidney transplant recipients. Finally, early cardiac graft outcomes (eg, requirements for VA-ECMO, rate of severe primary graft dysfunction) were not available during the study period, which is expected to be analyzed in the future. Finally, we have not looked at the outcomes of patients who were listed for SHKT but did not receive simultaneous kidney transplants.

## CONCLUSIONS

SHKT using DCD donors, regardless of the DCD recovery method, yields comparable early and mid-term survival and kidney graft outcomes when compared with those from DBD donors. There was a significant regional and institutional variation in the number of DCD-SHKT cases. Although the present data reflect the early experience of DCD-SHKT in the United States, our study suggests that using DCD donors for SHKT has the potential to safely expand the donor pool without compromising transplant outcomes in this growing, high-risk population that may benefit from dual-organ transplantation.

## Supplementary Material


