# Supplementary material for: Simultaneous Heart and Kidney Transplantation Using Circulatory Death Donors: Are Kidney Graft Outcomes Comparable With Brain Death Donors?
Source: Transplant Direct. 2025 Sep 18;11(10):e1853. doi: 10.1097/TXD.0000000000001853 (PMC12448170; doi:10.1097/TXD.0000000000001853)
Supplement: Supplementary file 1 [file txd-11-e1853-s001.pdf]

Supplemental Figures

Figure S1. Density Plot of Time between Brain Death to Cross Clamp

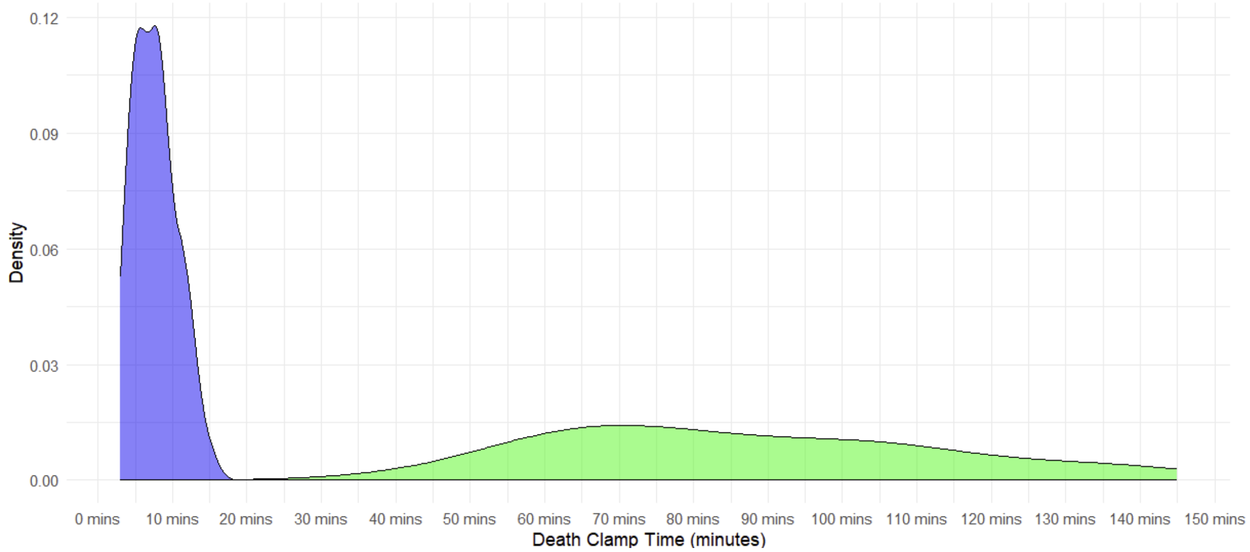

* Direct Procurement and Perfusion (DPP) was defined as 20 minutes or less from the time of brain death to the cross-clamp time. Normothermic Regional Perfusion (NRP) was defined as more than 20 minutes from the time of brain death to the cross-clamp time. Out of 91 DCD patients, 8 were excluded due to missing brain death times

Figure S2. Standardized Mean Differences of Covariates after Propensity Score Matching

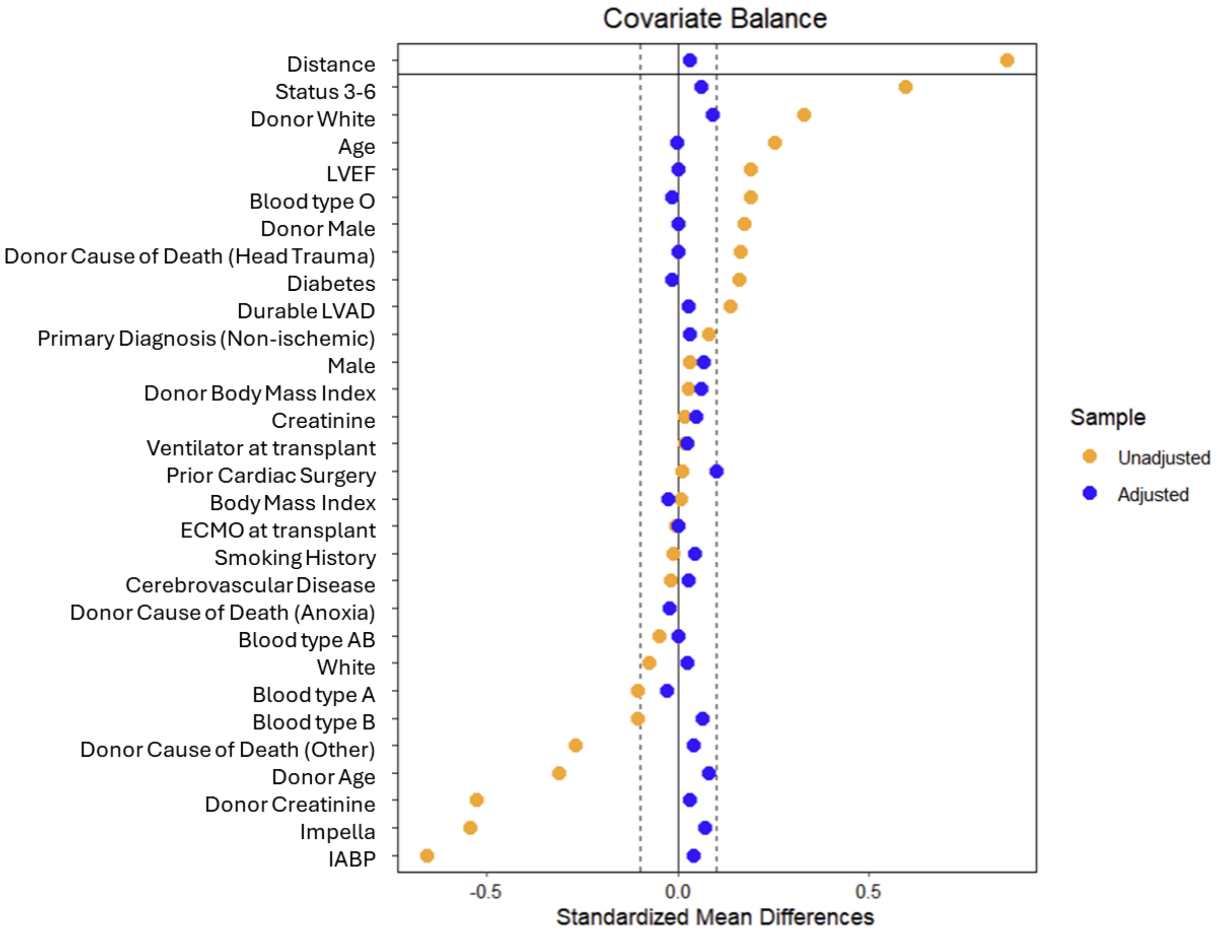

*Missing baseline data included recipient body mass index (BMI) (0.09%), donor BMI (0.09%), donor left ventricular ejection fraction (LVEF) (0.09%), HLA mismatch (1.2%), KDPI (10.4%), initial EPTS (2.3%), and end status of kidney (8.5%) in the unmatched cohort comparison, marked as unknown. Ethnicity/race was categorized as white vs. non-white; end status in the heart transplant waiting list was categorized as status 1 to 2 vs. status 3 to 6; the primary diagnosis of heart failure was categorized as ischemic cardiomyopathy vs. non-ischemic cardiomyopathy. Durable left ventricular assist device (LVAD) was defined as HeartMate II and III and HeartWare. Donor cause of death was summarized head trauma vs other. Among outcome variables, there were missing length of stay after heart transplant (1.8%), days on kidney waiting list (8.5%), acute rejection of kidney prior to discharge (8.7%), cold ischemic time of kidney (10.5%), and serum creatinine at discharge (8.7%). Only cases with complete baseline characteristics were used to perform propensity score matching, excluding 3 cases of DBD.

Considering the significantly small number of DCD cases, we used a 1:3 propensity score matching with the nearest neighbor using 17 recipient characteristics [age, gender, ethnicity/race, diabetes, BMI, smoking, creatinine, antibiotics use within 2 weeks prior to transplant, ischemic cardiomyopathy, use of Impella, durable LVAD and intra-aortic balloon pump (IABP), ventilator support, extracorporeal membrane oxygenation (ECMO), prior cardiac surgery, end status, and blood type] and 7 donor characteristics [age, gender, ethnicity/race, BMI, creatinine, cause

of death, and LVEF]. Covariates were selected based on clinical relevance and previous literature as described above.

Figure S3. Distribution of Initial EPTS and KDPI Score by Group

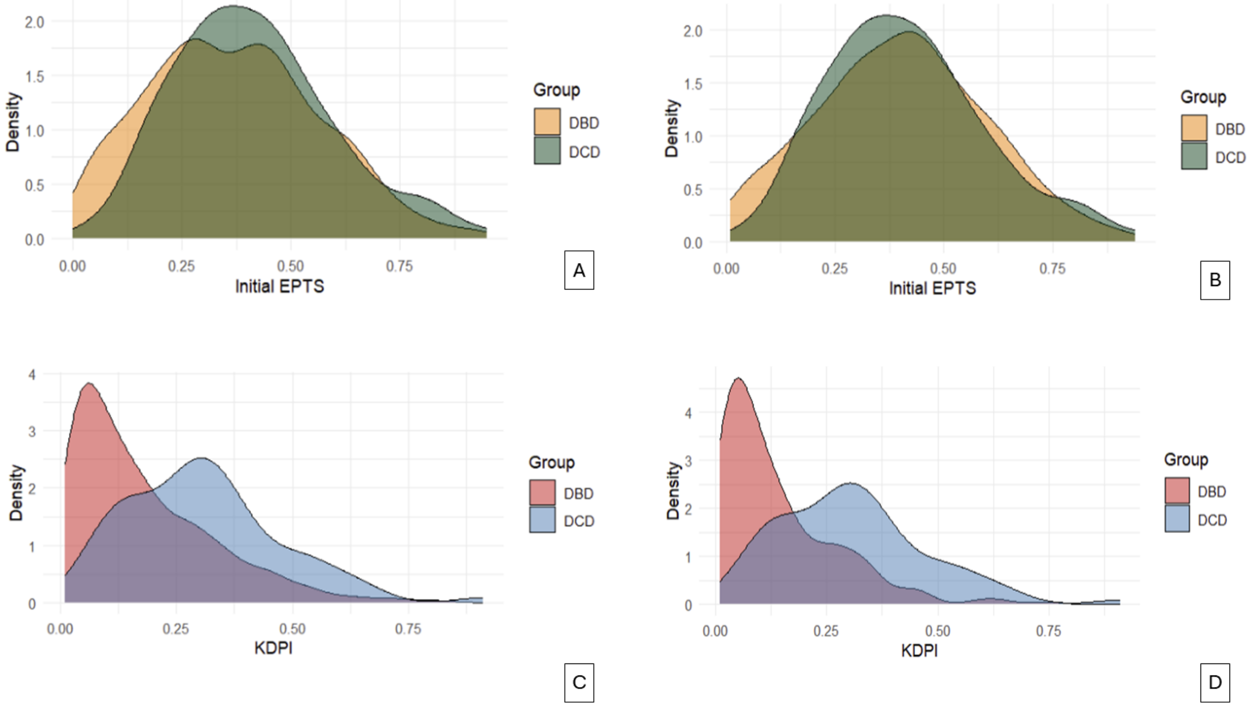

* Initial EPTS score: A; before matching, B; after matching, and KDPI score: C; before matching, D; after matching showed that despite higher scores for both initial EPTS and KDPI in the DCD cohort, the transplant outcomes were similar.

Figure S4. Recipient and Graft survivals outcome in Sub-analysis of Centers Performing Both DCD and DBD SHKT: DBD-SHKT vs. DCD-SHKT

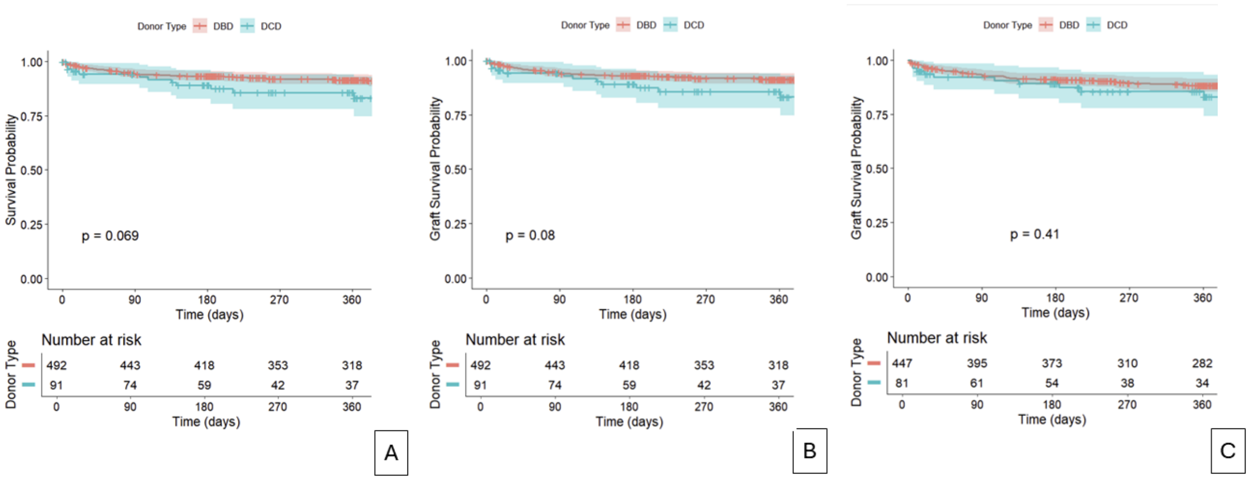

A; Recipient survival, B; Heart graft survival, and C; Kidney graft survival

Figure S5. Recipient and Graft survivals outcome in Sub-analysis between Centers Performing Both DCD and DBD SHKT (Center A) vs. Centers Performing only DBD SHKT (Center B)

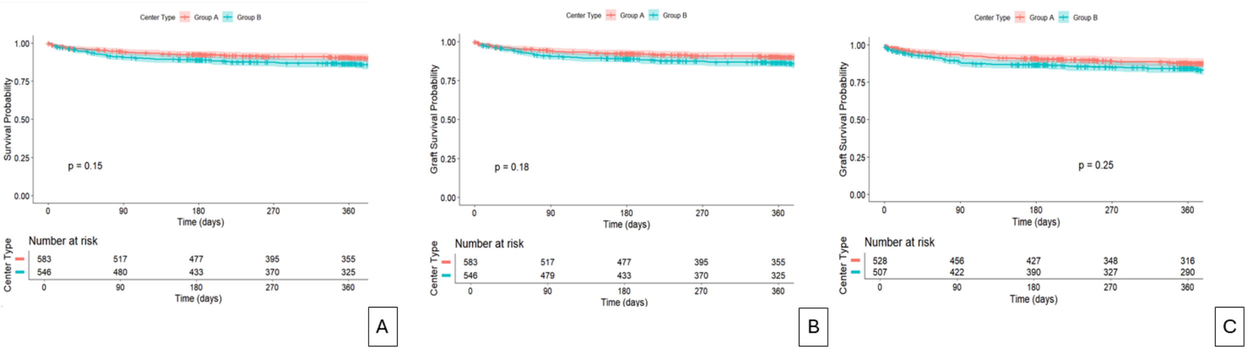

A; Recipient survival, B; Heart graft survival, and C; Kidney graft survival

Supplemental Tables

Table S1. Encrypted Transplant Centers Performing Both DCD and DBD SKHT

Transplant Center - Encrypted	Region	DBD-SHKT, N = 492[1]	DCD_SHKT, N = 91[1]	DCD-SHKT/Total SHKT ratio
1A	1	5 (1.0%)	8 (8.8%)	61.5%
1B	1	9 (1.8%)	2 (2.2%)	18.2%
1C	1	9 (1.8%)	3 (3.3%)	25%
2A	2	3 (0.6%)	2 (2.2%)	40%
3A	3	5 (1.0%)	4 (4.4%)	44.4%
3B	3	13 (2.6%)	1 (1.1%)	7.7%
3C	3	20 (4.1%)	3 (3.3%)	13.0%
3D	3	13 (2.6%)	1 (1.1%)	7.1%
4A	4	3 (0.6%)	3 (3.3%)	50%
4B	4	20 (4.1%)	2 (2.2%)	9.1%
4C	4	14 (2.8%)	1 (1.1%)	6.7%
5A	5	17 (3.5%)	14 (15%)	45.2%
5B	5	83 (17%)	1 (1.1%)	1.2%
5C	5	17 (3.5%)	3 (3.3%)	15%
6A	6	15 (3.0%)	2 (2.2%)	11.8%
7A	7	19 (3.9%)	1 (1.1%)	5%
7B	7	29 (5.9%)	1 (1.1%)	3.3%
7C	7	8 (1.6%)	1 (1.1%)	11.1%
7D	7	11 (2.2%)	1 (1.1%)	8.3%
7E	7	20 (4.1%)	2 (2.2%)	9.1%
8A	8	3 (0.6%)	1 (1.1%)	25%
8B	8	9 (1.8%)	1 (1.1%)	10%
8C	8	6 (1.2%)	1 (1.1%)	14.3%
9A	9	33 (6.7%)	2 (2.2%)	5.7%
9B	9	17 (3.5%)	3 (3.3%)	15%
10A	10	11 (2.2%)	2 (2.2%)	15.4%
11A	11	10 (2.0%)	1 (1.1%)	9.1%
11B	11	20 (4.1%)	12 (13%)	37.5%
11C	11	15 (3.0%)	1 (1.1%)	6.3%
11D	11	35 (7.1%)	11 (12%)	23.9%

* Encrypted Center Code was replaced by region and alphabet (e.g., region 1 center A – 1A, region 3 center C – 3C). DCD-SHKT/Total SHKT ratio shows proportion of SHKT out of total SHKT volume. For example, transplant center 1A had 61.5% DCD-SHKT/Total SHKT ration which means 61.5% of SHKT at center 1A was DCD-SHKT during the study period.

Table S2. Multivariable Cox Proportional Hazards Analysis for Recipient Mortality

	HR	Lower 95% Cl	Upper 95% Cl	p-value
Donor Type (DCD)	1.69	0.90	3.16	0.103
Recipient Age	1.02	1.01	1.04	0.012*
Recipient Gender (Female)	1.76	1.20	2.57	0.004*
Recipient Ethnicity/Race (White)	1.25	0.89	1.75	0.198
Dialysis at transplant	1.48	1.05	2.08	0.024*
IV antibiotics at transplant	1.36	0.88	2.09	0.166
Primary Diagnosis (Ischemic cardiomyopathy)	1.21	0.85	1.70	0.288
Durable LVAD (Yes)	1.41	0.91	2.20	0.122
IABP (No)	1.17	0.77	1.78	0.474
End Status (Status 1-2)	1.14	0.77	1.70	0.510
Donor Age	1.00	0.99	1.02	0.851
Donor Ethnicity/Race (White)	1.14	0.81	1.60	0.453
Donor Serum Creatinine	0.94	0.73	1.22	0.657
Center Type (Group B)	1.36	0.96	1.92	0.084

* Donor Type (DCD vs. DBD), LVAD=Left Ventricular Assist Device, IABP= Intra-Aortic Balloon Pump, Center Type (Group A; centers performed both DCD and DBD SHKT vs. Group B; centers performed only DBD SHKT)

Table S3. Baseline Characteristics and Transplant Outcomes of DCD-SHKT by Procurement Methods: DPP vs. NRP

Variable	DPP, N = 51[1]	NRP, N = 32[1]	p-value[2]
Baseline Characteristics			
Age, years	59.0 (52.5-63.0)	63.5 (59.0-67.0)	0.010
Male	39 (76%)	29 (91%)	0.10
Race			0.16
Non-White	35 (69%)	17 (53%)	
White	16 (31%)	15 (47%)	
Diabetes mellitus	31 (61%)	16 (50%)	0.33
BMI, kg/m^2	27.3 (24.0-[illegible]	26.8 (24.5-[illegible]	0.56
Dialysis at transplant	13 (25%)	10 (31%)	0.57
History of smoking	24 (47%)	10 (31%)	0.15
Cerebrovascular disease	3 (5.9%)	1 (3.1%)	>0.99
Infection requiring IV drug therapy	4 (7.8%)	1 (3.1%)	0.64
Heart Transplant End Status			0.33
Status 1-2	23 (45%)	11 (34%)	
Status 3-6	28 (55%)	21 (66%)	
Heart Failure Primary Diagnosis			0.44
Ischemic CM	15 (29%)	12 (38%)	
Non-Ischemic	36 (71%)	20 (63%)	
Durable LVAD	13 (25%)	3 (9.4%)	0.070

IABP	5 (9.8%)	2 (6.3%)	0.70
Impella	0 (0%)	1 (3.1%)	0.39
Ventilator	2 (3.9%)	0 (0%)	0.52
ECMO	4 (7.8%)	1 (3.1%)	0.64
History of cardiac surgery	18 (35%)	8 (25%)	0.33
Initial EPTS score	36 (28-46)	46 (31-50)	0.024
Blood Type			0.35
A	14 (27%)	13 (41%)	
AB	2 (3.9%)	2 (6.3%)	
B	9 (18%)	2 (6.3%)	
O	26 (51%)	15 (47%)	
Donor age, years	28.0 (21.0-34.0)	33.0 (28.5-39.0)	0.002
Donor male	39 (76%)	31 (97%)	0.013
Donor Race			0.11
Non-White	11 (22%)	12 (38%)	
White	40 (78%)	20 (63%)	
Donor BMI, kg/m^2	26.8 (23.6-31.6)	28.7 (24.7-32.2)	0.47
Donor Creatinine	0.7 (0.6-0.9)	0.8 (0.7-1.0)	0.007
Donor Cause of Death (Head Trauma)	23 (45%)	18 (56%)	0.42
Donor LVEF, %	64.0 (60.0-65.0)	65.0 (58.0-70.0)	0.56
Donor KDPI score	19 (8-28)	27 (23-38)	0.004

Distance, miles	400.0 (188.0-619.0)	222.5 (59.3-392.3)	0.021
Days in Waiting list, days	54.0 (23.0-239.0)	23.5 (6.8-83.8)	0.075
Heart Ischemic Time, hours	5.9 (4.8-6.8)	3.6 (2.9-4.1)	<0.001
Kidney Cold Ischemic Time, hours	19.1 (12.9-24.1)	19.3 (16.4-27.0)	0.52
Kidney Waiting list Time, days	44.0 (15.0-221.8)	27.0 (9.0-165.5)	0.39
Kidney Status at Transplant			0.24
Active	48 / 48 (100%)	25 / 27 (93%)	
Temporarily Inactive	0 / 48 (0%)	2 / 27 (7.4%)	
Kidney Side, Lt	39 / 48 (81%)	18 / 27 (67%)	0.26
Transplant Outcomes			
Acute Rejection	4 (7.8%)	1 (3.1%)	0.69
Dialysis	21 (41%)	10 (31%)	0.50
Stroke	1 (2.0%)	1 (3.1%)	>0.99
Pacemaker	0 (0%)	2 (6.3%)	0.28
Heart Transplant Length of Stay	22.0 (16.3-34.0)	18.0 (12.0-31.0)	0.23
Patient Mortality at 30 Days	3 (5.9%)	1 (3.1%)	0.96
Patient Mortality at 6 months	4 (7.8%)	4 (13%)	0.75
Heart Graft Failure at 6 months	4 (7.8%)	4 (13%)	0.75
Heart Graft Failure at 1 year	6 (12%)	5 (16%)	0.86
Kidney Graft Failure at 6 months	5 (10%)	2 (7.4%)	0.99

Kidney Graft Failure at 1 year	7 (15%)	3 (11%)	0.94
Kidney Acute Rejection prior to discharge	0 (0%)	1 (3.7%)	0.77
Kidney Delayed Graft Function	15 (29%)	7 (22%)	0.63
Unknown	3 (5.9%)	5 (16%)	
Resume Dialysis Maintenance after KI transplant	1 (2.0%)	0 (0%)	>0.99
Recipient creatinine at Discharge	2.0 (1.3-3.1)	1.3 (1.1-1.8)	0.008

* Direct Procurement and Perfusion (DPP) was defined as 20 minutes or less from the time of brain death to the cross-clamp time. Normothermic Regional Perfusion (NRP) was defined as more than 20 minutes from the time of brain death to the cross-clamp time. Out of 91 DCD patients, 8 were excluded due to missing brain death times

Table S4. Recipient, Donor, and Organ Procurement Characteristics and Outcomes of Sub-group Cases at Centers Performed Both DCD and DBD SHKT.

Variable	DCD-SHKT, N = 91[1]	DBD-SHKT, N = 492[1]	p-value[2]
Baseline Characteristics			
Age, years	60.0 (53.0-65.5)	58.0 (50.8-64.0)	0.054
Male	74 (81%)	390 (79%)	0.76
Race			0.49
Non-White	55 (60%)	275 (56%)	
White	36 (40%)	217 (44%)	
Diabetes mellitus	50 (55%)	226 (46%)	0.14
BMI, kg/m^2	27.0 (24.0-31.6)	27.1 (23.8-30.8)	0.94
Dialysis at transplant	25 (27%)	196 (40%)	0.034
History of smoking	36 (40%)	209 (42%)	0.69
Symptomatic cerebrovascular at registration	8 (8.8%)	45 (9.1%)	>0.99
Infection requiring IV drug therapy	6 (6.6%)	77 (16%)	0.035
Status			<0.001
Status 1-2	39 (43%)	333 (68%)	
Status 3-6	52 (57%)	159 (32%)	
Primary Diagnosis			0.61
Ischemic CM	28 (31%)	168 (34%)	
Non-Ischemic CM	63 (69%)	324 (66%)	
Durable LVAD	17 (19%)	46 (9.3%)	0.014

IABP	8 (8.8%)	136 (28%)	<0.001
Impella	1 (1.1%)	25 (5.1%)	0.16
Ventilator support	2 (2.2%)	13 (2.6%)	>0.99
ECMO	5 (5.5%)	21 (4.3%)	0.81
History of cardiac surgery	31 (34%)	165 (34%)	>0.99
Initial EPTS score	41 (28-51)	35 (22-51)	0.077
Recipient ABO			0.12
A	30 (33%)	201 (41%)	
AB	4 (4.4%)	25 (5.1%)	
B	12 (13%)	88 (18%)	
O	45 (49%)	178 (36%)	
Donor age, years	30.0 (24.0-35.0)	33.0 (25.0-41.0)	0.002
Donor male	76 (84%)	372 (76%)	0.13
Donor race			0.025
Non-White	24 (26%)	194 (39%)	
White	67 (74%)	298 (61%)	
Donor BMI, kg/m^2	27.1 (24.3-31.6)	26.8 (23.6-31.1)	0.44
Donor Creatinine, mg/dl	0.7 (0.6-1.0)	0.9 (0.7-1.2)	<0.001
Donor Cause of Death			0.10
Head Trauma	46 (51%)	200 (41%)	
Other	45 (49%)	292 (59%)	
Donor LVEF	64.0 (60.0-[illegible]	60.0 (57.0-[illegible]	0.10

Donor KDPI score	22 (13-32)	20 (9-35)	0.49
ABO match			0.50
Identical	79 (87%)	410 (83%)	
Compatible	12 (13%)	82 (17%)	
Transplant Outcomes			
Procurement Distance, miles	344.0 (153.5-581.5)	231.5 (76.8-407.0)	<0.001
Heart Waiting list Time, days	42.0 (9.5-160.5)	27.5 (9.0-85.3)	0.071
Kidney Waiting list Time, days	37.0 (10.0-164.0)	22.0 (8.0-77.0)	0.011
Heart Ischemic Time, hours	4.9 (3.4-6.4)	3.5 (2.9-4.0)	<0.001
Heart Machine Perfusion	53 / 91 (58%)	30 / 492 (6.1%)	<0.001
Kidney Cold Ischemic Time, hours	19.2 (13.7-24.8)	14.4 (8.5-20.5)	<0.001
Acute Rejection	6 / 91 (6.6%)	32 / 492 (6.5%)	>0.99
Dialysis	34 / 91 (37%)	172 / 490 (35%)	0.77
Stroke	2 / 91 (2.2%)	33 / 488 (6.8%)	0.15
Pacemaker	2 / 91 (2.2%)	5 / 490 (1.0%)	0.67
Heart Transplant Length of Stay	20.0 (15.0-34.0)	19.0 (14.0-30.0)	0.29
Patient Mortality at 30 Days	5 / 91 (5.5%)	14 / 492 (2.8%)	0.32
Patient Mortality at 6 months	9 / 91 (9.9%)	32 / 492 (6.5%)	0.35
Heart Graft Failure at 6 months	9 / 91 (9.9%)	33 / 492 (6.7%)	0.39

Heart Failure at 1 year	12 / 91 (13%)	40 / 492 (8.1%)	0.18
Heart Graft Failure at 6 months	8 / 81 (9.9%)	38 / 447 (8.5%)	0.85
Kidney Graft Failure at 1 year	11 / 81 (14%)	48 / 447 (11%)	0.58
Kidney Acute Rejection prior to discharge	1 / 81 (1.2%)	7 / 447 (1.6%)	>0.99
Kidney Status at Transplant			>0.99
Active	79 / 81 (98%)	439 / 447 (98%)	
Temporarily Inactive	2 / 81 (2.5%)	8 / 447 (1.8%)	
Kidney Side, Left	62 / 81 (77%)	316 / 447 (71%)	0.35
Kidney Delayed Graft Function	25 / 91 (27%)	132 / 492 (27%)	0.81
Resume Dialysis Maintenance after KI transplant	1 / 91 (1.1%)	11 / 492 (2.2%)	0.76
Recipient creatinine at Discharge	1.6 (1.2-2.4)	1.3 (0.9-1.9)	<0.001

Table S5. Recipient, donor, and organ procurement characteristics and outcomes between centers that performed both DCD and DBD SHKT (DCD-SHKT centers) versus centers that performed only DBD SHKT (Non DCD-SHKT centers)

Variable	DCD-SHKT centers, N = 583	Non DCD-SHKT Centers, N = 546	p-value
Baseline Characteristics			
Recipient Age	58.0 (51.0-64.0)	58.0 (50.3-64.0)	0.30
Male	464 (80%)	442 (81%)	0.62
Race			0.85
Non-White	330 (57%)	313 (57%)	
White	253 (43%)	233 (43%)	
Diabetes	276 (47%)	259 (47%)	>0.99
BMI	27.1 (23.9-30.9)	27.4 (24.0-31.1)	0.64
Dialysis at transplant	221 (38%)	219 (40%)	0.49
History of smoking	245 (42%)	204 (37%)	0.12
Symptomatic cerebrovascular at registration	53 (9.1%)	51 (9.3%)	0.97
Infection requiring IV drug therapy	83 (14%)	82 (15%)	0.77
Status			<0.001
Status 1-2	372 (64%)	418 (77%)	
Status 3-6	211 (36%)	128 (23%)	
Primary Diagnosis			0.68
Ischemic CM	196 (34%)	191 (35%)	
Non-Ischemic	387 (66%)	355 (65%)	
Durable LVAD	63 (11%)	92 (17%)	0.004
IABP	144 (25%)	149 (27%)	0.36
Impella	26 (4.5%)	45 (8.2%)	0.013
Ventilator	15 (2.6%)	8 (1.5%)	0.27
ECMO	26 (4.5%)	37 (6.8%)	0.12
History of cardiac surgery	196 (34%)	181 (33%)	0.92
EPTS score	36 (23-51)	36 (22-49)	0.19
Recipient ABO			0.33
A	231 (40%)	194 (36%)	
AB	29 (5.0%)	31 (5.7%)	
B	100 (17%)	86 (16%)	
O	223 (38%)	235 (43%)	
Donor age	32.0 (25.0-40.0)	31.0 (24.0-38.0)	0.11
Donor male	448 (77%)	429 (79%)	0.53
Donor race			0.13
Non-White	218 (37%)	229 (42%)	
White	365 (63%)	317 (58%)	
Donor BMI	26.8 (23.6-31.3)	27.3 (23.6-31.1)	0.73
Donor Creatinine	0.9 (0.7-1.2)	0.9 (0.7-1.2)	0.29
Donor Cause of Death			0.55
Head Trauma	246 (42%)	241 (44%)	
Other	337 (58%)	305 (56%)	
Donor LVEF	60.0 (57.0-65.0)	61.0 (57.0-65.0)	0.78
Donor KDPI score	21 (9-35%)	16 (7-28%)	<0.001
Procurement Distance (miles)	247.0 (82.5-	231.5 (107.5-	0.51
Heart Waiting list Time (days)	29.0 (9.0-90.5)	39.0 (14.0-144.3)	<0.001
Kidney Waiting list Time (days)	24.0 (8.0-87.3)	35.0 (12.0-111.0)	<0.001

Heart Ischemic Time (hours)	3.5 (3.0-4.1)	3.5 (2.9-4.1)	0.090
Heart Machine Perfusion	83 / 583 (14%)	14 / 546 (2.6%)	<0.001
Kidney Cold Ischemic Time (hours)	15.7 (8.7-21.2)	18.0 (11.4-22.9)	<0.001
Transplant Outcomes			
Acute Rejection of Heart	38 / 583 (6.5%)	57 / 546 (10%)	0.024
Dialysis	206 / 581 (35%)	193 / 545 (35%)	>0.99
Stroke	35 / 579 (6.0%)	11 / 543 (2.0%)	0.001
Pacemaker	7 / 581 (1.2%)	9 / 545 (1.7%)	0.70
Length of Stay	19.0 (14.0-31.0)	24.0 (17.0-36.0)	<0.001
Patient Mortality at 30 Days	19 / 583 (3.3%)	21 / 546 (3.8%)	0.71
Patient Mortality at 6 months	41 / 583 (7.0%)	59 / 546 (11%)	0.034
Patient Mortality at 1 year	51 / 583 (8.7%)	69 / 546 (13%)	0.043
Heart Graft Failure at 6 months	42 / 583 (7.2%)	59 / 546 (11%)	0.044
Heart Graft Failure at 1 year	52 / 583 (8.9%)	69 / 546 (13%)	0.055
Kidney Graft Failure at 6 months	46 / 528 (8.7%)	65 / 507 (13%)	0.042
Kidney Graft Failure at 1 year	59 / 528 (11%)	75 / 507 (15%)	0.10
Kidney Acute Rejection prior to discharge	8 / 528 (1.5%)	3 / 503 (0.6%)	0.26
Kidney End Status			0.27
Active	518 / 528 (98%)	489 / 505 (97%)	
Temporarily Inactive	10 / 528 (1.9%)	16 / 505 (3.2%)	
Kidney Side, Lt	378 / 528 (72%)	326 / 507 (64%)	0.014
Kidney Delayed Graft Function	157 / 583 (27%)	142 / 546 (26%)	0.54
Resume Dialysis Maintenance after KI	12 / 583 (2.1%)	22 / 546 (4.0%)	0.078
Recipient creatinine at Discharge	1.3 (1.0-2.0)	1.3 (0.9-1.8)	0.010
